# A cognitive task analysis of a visual analytic workflow: Exploring molecular interaction networks in systems biology

**DOI:** 10.5210/disco.v6i0.3410

**Published:** 2011-03-21

**Authors:** Barbara Mirel, Felix Eichinger, Benjamin J Keller, Matthias Kretzler

**Affiliations:** 1 School of Education, University of Michigan, Ann Arbor, Michigan, 48109United States; 2 Department of Internal Medicine, University of Michigan, Ann Arbor, Michigan, 48109United States; 3 Department of Computer Science, Eastern Michigan University, Ypsilanti, Michigan, 48197United States

**Keywords:** systems biology, usability, cognition, molecular interaction networks, visualization, human-computer interaction, translational medicine

## 1. Background

In bioinformatics the development of sufficiently robust and useful tools for exploratory analysis is in its infancy. Payne et al. argue that despite a general availability of applications the “absence of sufficiently robust analytical tools capable of addressing the requirements of specific research questions” significantly impedes translational research (1, 137). One reason for this problem is a paucity of knowledge about “the requirements of specific research questions” from the analytical perspectives of biomedical specialists. As requirements engineering experts emphasize, to be useful and sufficiently robust applications must fit domain specialists’ actual investigative approaches for specific research questions and goals (2). Our study assumes the perspective of a domain specialist and focuses on one common class of exploratory analysis in translational research with visualization tools. Specifically, we conducted a case-based, cognitive task analysis of a specialist’s workflow for functionally analyzing expression data in molecular interaction networks for hypothesizing purposes. The workflow also includes drawing relationships between molecular-based insights from network analysis and phenotype data. 

We constructed representations of the biomedical specialist’s ways of knowing and reasoning while visually analyzing graphics such as heat maps, interactive networks, and data tables. We also validated that our representations are at levels of detail appropriate for guiding development. Our study takes an initial step toward the long term bioinformatics goal of establishing cognitive-oriented user models to guide tool development for this workflow. 

From our analysis, we specify discernible inferences that mark progress toward generating a hypothesis, what we call milestone inferences. We identify these milestone inferences and trace them to the cognitive tasks leading up to them. We capture this workflow in complementary representations, which break new ground in the semantic specificity they provide for cognitive tasks in this complex analysis. Additionally, we demonstrate that the levels of detail in our representations can be applied to tool development and lead to a working prototype sensitive to scientists’ actual flow of cognitive tasks. 

Findings from our case provide initial steps toward modeling this workflow for the purpose of guiding development. As such they have preliminary implications for tool development. We propose several implications, including the need to develop features and functions in bundles that correspond to the individual and combined cognitive tasks that specialists enact during specific components (patterns) of their analysis. We also suggest certain priorities for visual analytic support that could help achieve sufficient robustness and usefulness. 

## 2. Relevant Research

Our study tackles two perennial challenges: (1) Achieving sufficient robustness and usefulness in tools for scientists’ exploratory analyses; and (2) representing analytical workflows at levels of detail that “speak to developers” for tool implementation. Currently, two fields of study are increasingly addressing these challenges – cognitively-oriented science studies and information visualization/visual analytics. In both fields researchers agree on broad terms to define processes of discovery, terms such as information foraging, sensemaking, knowledge construction, interpretation, inference, insight, and hypothesizing (4; 5). They also commonly study discovery processes from a distributed cognition perspective. Based on this perspective, cognition for software-supported discovery is distributed between human thinking and electronic workspaces in a dynamic system. That is, workspace affordances constrain scientists’ tasks and reasoning; and scientists’ goals, objectives, ways of knowing, reasoning, and time pressures constrain both the value scientists give to workspace features and functions and the actual use they make of them. Distributed cognition grounds our study, as well. 

Shared perspectives across science studies and information visualization are important but they alone cannot overcome the first challenge mentioned previously – i.e., developing sufficiently useful tools for discovery-driven analysis. In both fields, more research is needed at a concrete, application level in terms of support that can adequately “connect user objectives with the [tool] interaction techniques that help accomplish them” (6, 1225). This application level of understanding is required for mapping scientists’ complex analysis processes to tool requirements. 

In science studies, researchers get somewhat concrete by defining the higher order thinking implicit in discovery-driven analysis. They define it as a mix of reasoning modes, including classification, comparison, mental modeling, story-telling, validation, and metacognition (i.e. reasoning to monitor and manage inquiry) (7). These investigators highlight, as well, that throughout analysis scientists recursively construct and apply certain patterns of multi-modal reasoning (7). For their part, information visualization researchers concretely characterize discovery-driven reasoning as interactions called “thinking tasks” that can be tied to users’ analytical intentions (8). An example is “find items with a given attribute.” These task types are generic and serve the purpose of populating taxonomies (9; 10). Despite the beginnings of concrete characterizations by investigators in both fields, representations of discovery-driven reasoning and tasks are still too generic to guide development. Moreover, they often are piecemeal - separated from a full coherent flow of analysis; and for our targeted workflow, they are far too incomplete.

Fortunately, some general patterns have been uncovered in bioinformatics for this targeted workflow. They include scientists’ ways of arranging and rearranging data and relationships to classify by biological traits, and scientists’ processes of overlaying differential expression values onto pathways associated with molecular interactions of interest (11-15). Bioinformatics studies also reveal that for the workflow we target scientists often use separate tools for different parts of the workflow but still consider it one unified flow (16). Our cognitive task analysis shows this flow performed in different tools, but our findings suggest that visualization tools could be designed to include affordances for more parts of the flow or to better integrate work across tools if they were more attuned to scientists’ actual *compositional* reasoning and actions.

The second challenge that our study tackles - representing analytical workflows at levels of detail that “speak to developers” – is confounding because researchers within and across fields do not share common meanings of complex discovery processes or define them at the same level of detail. For example, Chang et al. keep the conversation broad by defining “insight” as “knowledge building” (17). Saraiya et al. seem to get more precise by defining insight as “an individual observation about the data by a participant, a unit of discovery” (15, 444). Yet they leave vague what “data observation” and “discovery” are. For example, do these processes involve literally reading data off a display, interpreting, inferring, or all three? 

In cognitively-oriented science studies, Trickett and Trafton help clarify what is meant by deep insight and the types of human knowledge processing it demands (18). They argue that literally reading explicit information off displays - e.g. values, patterns, or labels - is not enough to generate the deep insights that lead to hypotheses. In addition to read-offs, scientists have to be able to spatially transform explicitly displayed data and views into implicit information and relevant meanings. In these transformations, scientists modify data in external views and spatially manipulate the views and relationships both externally and mentally to gain a different understanding. Trickett and Trafton underscore that scientists must combine reasoning *and* transformative actions to gain insights and inferences.

To summarize, this review shows that the current research relevant to modeling analysts’ workflows for the translational research that we target is broad and generically concrete but not domain- or problem-specific enough for development purposes. Largely, it provides just an outline of discovery-driven workflows. We contend that this outline needs to be filled out by addressing the following questions:

What analytical objectives structure this flow of knowledge work?
What components of core knowledge work – as scientists define these components – combine to achieve each objective?
What interwoven cognitive tasks make up each component of core knowledge work?
What (milestone) inferences are derived from goal-specific components and their constituent cognitive tasks that are critical to moving closer to generating a hypothesis?
What mappings might be made between cognitive tasks associated with milestone inferences and visualization requirements that could improve fitness to purpose in tools?

**Figure 1.**
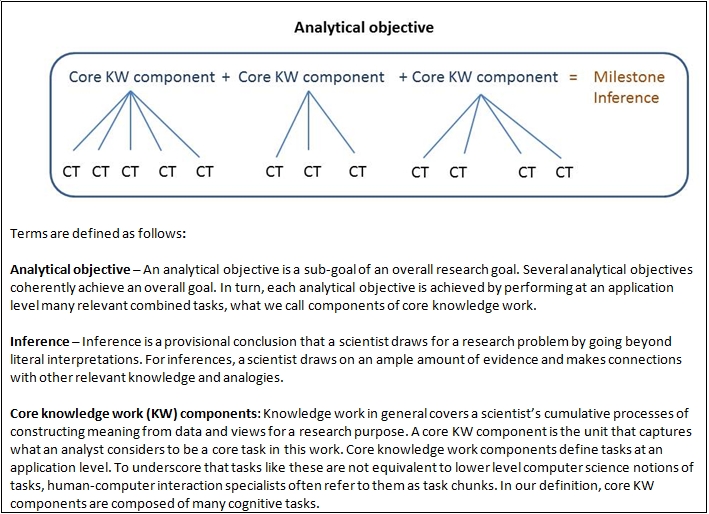
Application level template or describing the tiers of workflow performance.

Our case-based, cognitive task analysis is designed to address these questions. As the background literature suggests, addressing them involves engaging discovery-related concepts that in themselves are differently understood within and across fields. We explicate our terms as they apply to the workflow we target in the Methods section, Figure 1.

## 3. Methods

To address the questions above amid sparse prior research, we conducted a case-based, cognitive task analysis as an appropriate initial step (19). As qualitative methodologists advise, we grounded our methods in an empirically-based understanding of scientists’ situated patterns of cognitive and behavioral discovery-driven analysis for similar systems biology problems drawn from the research literature (20). For our case study, we focused on the work conducted by one of the co-authors (FE), a biomedical specialist with extensive experience in the translational research workflow that we target. We refer to him throughout as the “biomedical specialist.” In this section we describe the workflow site and research problem and then detail the methods of our four-phase cognitive task analysis. 

### 3.1. Study site 

The biomedical specialist in the case has many years of experience and success working in an internationally renowned renal disease laboratory that has made considerable progress in Type 1 (T1) translational research. T1 research - “bench to bedside” - aims to move basic research discoveries from the laboratory to clinical practice by taking a systems approach to analyzing molecular and clinical data. This laboratory is a model of trans-disciplinary teamwork for translational research. Specialists from multiple disciplines sit side-by-side – bench scientists, statisticians, informatics experts, and clinical researchers – and collaborate on research project teams. Clinicians also actively participate in research teams. As an effective means for bridging bench findings (e.g. on disease versus healthy tissue samples and high throughput analyses) to clinical (phenotype) data and clinical implications (e.g. treatments), multidisciplinary research processes in the laboratory are modular. That is, specialists in each project have prime responsibility for delving into their respective areas of expertise, and they collaboratively discuss and review their work continuously with their teammates from other specializations. 

The biomedical specialist in our case is an informatics expert who explores biological not computational problems for translational renal disease research. He conducts the software-mediated workflow we study with a variety of tools and numerous collaborations. He collaborates with bench scientists and biostatisticians on his project team as they process, format, and group experimental data for his investigation. He then delves into the targeted workflow with visualization applications. He is an expert in inferring meaning from graphic structures such as heat maps and networks. As he notes, visual analytics parade his perception, higher order cognition, and cognitive task flows. He remarks that he is “agnostic to the genes that biological researchers love;” and he turns to his collaborators for domain content at various points in his analysis. 

As findings emerge from his visual analytics he collaborates mainly with two clinical researchers, one of whom is co-author MK, the laboratory director. Together, they share insights, test them against biological and clinical expertise, and determine next investigative steps. Overall, the processes structuring this workflow exemplify translational research processes advocated in the research literature (21).

### 3.2. Translational research problem and workspaces

The workflow in the case is part of a larger translational research project in this renal disease laboratory, a project aimed at uncovering molecular influences that suggest ways to classify renal sub-diseases genotypically. Ultimately, the research will lead to more precise, mechanistic definitions that can improve screening, diagnosis, and treatment. Data in the case come from biopsy samples from 250 renal disease patients for 13 clinically-defined sub-diseases and samples from a control (healthy) group of live donors. In the workflow that we study the biomedical specialist starts with 12,000 genes from microarray analysis. His laboratory team shares some initial mental models of possible molecular mechanisms of sub-diseases. However, the biomedical specialist’s data-driven, structural analysis of molecular interaction networks will push the team’s understanding farther. 

The workspaces afforded by visualization software also shape how the biomedical specialist performs this workflow. The software helps the biomedical specialist functionally analyze differentially expressed genes by bringing in information from publicly available databases on gene attributes, protein-protein interactions, pathways, and knowledge derived from text mining and statistical analysis. The biomedical specialist uses different tools for distinct parts of the workflow. He has attempted at times to conduct this workflow in a single network visualization tool but has had to undertake a lot of scripting and workarounds without achieving adequate analytical success. He has not yet found a single tool robust enough for his hypothesizing objectives and analytical practices. 

### 3.3. Cognitive task analysis

The cognitive task analysis was conducted by BM, the lead author and a visualization and human-computer interaction specialist. The cognitive task analysis includes iterative participatory feedback from the biomedical specialist. This analysis had four phases, as follows.

#### Phase 1: Analysis of preliminary knowledge

In the first phase, the co-author BK, a researcher in computer science and software engineering, collaborated with BM to review data from Mirel’s prior field study of the same biomedical research problem and other relevant research (11; 23; 24). The aim was to identify issues that the cognitive task analysis needed to address. The review showed the paucity of application-level knowledge for representing a coherent and complete workflow in exploratory analysis of molecular networks. Also, the review demonstrated that in conducting this workflow scientists commonly falter due to mismatches between their needs and tool capabilities when moving from characterizing molecular relationships descriptively to explaining their possible functional roles contextually in a disease. We, therefore, decided to focus our case on a biomedical specialist’s incremental processes of moving toward explanatory inferences during the targeted workflow so that our study would address a salient aspect of complex analysis in need of better tool support. 

We determined that the scope of the workflow would span from a biomedical specialist’s initial inquiry into functional relationships associated with differentially expressed genes to the point at which the specialist had drawn sufficient inferences about potentially influential molecular relationships in disease mechanisms. At this point, the specialist’s would be ready to advance to the next phase of analysis. The next phase, a different workflow, involves investigating in more depth causal and conditional explanations. Prior research by Mirel and others reveals that after specialists develop initial inferences about promising molecular relationships they advance to this next phase (11; 13; 25; 26). In this next phase, scientists examine subsystems of functional relationships in biological contexts, inquire into biological events, and engage in computational modeling and/or predictive analysis. The endpoint of the workflow we study occurs when the specialist is confident enough in his provisional explanatory inferences to move to this next workflow. 

#### Phase 2: Focused knowledge elicitation 

To elicit knowledge from the biomedical specialist, interview probes inquired into his underlying analytical questions, strategies, objectives, rationales for various analytical choices, and ways of thinking and knowing. Interview questions also elicited details about analytical processes and interactions while collaborating with clinical colleagues to explore relevant biological meanings.

#### Phase 3: Iterative construction of representations. 

BM cross-referenced interview findings to the research literature to assure that issues found in the interviews corresponded with a larger population’s activities, and to highlight open questions in both the research literature and interview data. BM, FE, and BK collaborated at different times and in varying degrees to determine how to represent the workflow. To our knowledge, no current studies trace a scientist’s flows of reasoning-and-action to inferences relevant to hypothesizing for this type of visual analytic workflow. Consequently, an established structure does not exist for organizing and representing case-based renditions of the workflow. In charting this new territory, one of our principal criteria was that levels of granularity and coherence in the representations had to do justice to the complexity of the work while still being able to speak to tool developers. That is, representations could be neither so low level (programmatic) to elude the adaptations scientists enact in this intellectually complex analysis nor so ethnographically thick to defy ready translation to tool requirements.

To address this challenge, we alternately tried structuring workflow descriptions by the tools used, by the types of data analyzed, by the types of visualizations, and by problem-solving tasks. We settled on problem-solving tasks. To arrive at this decision BM wrote and rewrote scenarios from different angles, each accommodating a different way of structuring our data analysis. We found that structuring representations by problem-solving objectives was the only option that let us trace a flow of software-mediated reasoning-and-action toward inference with the necessary coherence, completeness, and accuracy. Moreover, a problem-solving logic seemed to synthesize the reasoning and task orientations that investigators in science studies and information visualization/visual analytics respectively take to characterize scientists’ tasks. 

In the problem-solving orientation, we structured the flow of analysis in three tiers. (See Figure 1 for a template of these tiers and an explication of our terms.) At the top tier are the analytical objectives that drove the biomedical specialist’s investigation into his research problem. In the next tier, we abstracted the biomedical specialist’s conception of the core knowledge work he enacted to perform each objective and called each core task a core knowledge work component. In the third tier, we identified the cognitive tasks constituting the performance of each of these components of core knowledge work for each objective. 

In addition to representing the workflow in a problem-solving scenario, we also represented it in flow diagrams that capture the actual work conducted in these tiers. We created a third type of representation, as well - a taxonomy of higher order reasoning/cognitive tasks organized by mode of reasoning (see Supplemental Material 1). These three forms of representation should resonate with the development community. Scenario-based usage cases exemplify a cognitive engineering approach that has proven successful in modeling complex work for development purposes; workflow diagrams are a common means for depicting user tasks for tool development (27; 28); and task taxonomies are common in information visualization (10). A fourth representation emerged, as well, during Phase 4.

#### Phase 4: Validation of representations for translating to tool development

To assure that our three complementary representations of the workflow could guide tool development toward usefulness, BM worked with FE and, in early versions with BK, to create a trial mapping of cognitive tasks to user requirements for visual analytic support. We focused on only a small subset of cognitive tasks – those composing the core knowledge work for one mini-flow within this workflow. This mini-flow related to the biomedical specialist’s activity of classifying like with like by selecting and grouping relationships based on two or more similar traits. To map this subset of cognitive tasks to user-centered visualization requirements, we synthesized relevant requirements from the research literature across many related disciplines. This approach assured that any requirements we mapped had already been vetted for tasks similar to those we selected to map - though not always implemented. 

As in other compilations of visualization requirements in the research literature our methods for identifying relevant requirements were informal yet well-grounded for our purposes (29). We examined research from diverse relevant disciplines - visual analytics, usability, human-computer interaction, information visualizations, and cognitive science. We chose only research aimed at audiences concerned about designing, developing, and/or evaluating visualization applications for usefulness and usability. Articles aimed at these audiences and purposes offered requirements framed in a language and level of detail meaningful to tool development purposes. Sources for our requirements were not exhaustive but were comprehensive enough to provide numerous relevant findings for the selected cognitive tasks, and to prove feasibility for this mapping technique. One criterion for selecting articles was that relevant articles had to have some component of user-based evaluation or real world application. Additionally, requirements had to target cognitively demanding work and needed to be justified according to user/task need and/or visual theory. We did not include requirements targeting low level operations but instead included such terms as “efficiently” or “quickly” in the requirements statements we composed to imply the need for optimal low level operations. Moreover, we largely focused on research published after 2002. Most of the requirements were found in more than one reference source. The Supplemental Material 2 presents the requirements and their references. 

We removed redundancies from the requirements we collected and clarified and assured consistency in language. We then mapped them to the select cognitive tasks. We tested the feasibility of using the mapping for tool development by working with a senior developer to design and develop a prototype of a visualization plugin based on the bundles of requirements represented in the mapping. Because our focus is proving feasibility we did not test the mappings for reliability or generalizability. 

## 4. Results

Results from our study fill out the current outline of biomedical researchers’ discovery-driven exploratory analysis of molecular interaction networks. Below we narrate and diagram the analysis, and organize it by the biomedical specialist’s three objectives online and one offline objective (which has no diagramming). The objectives and associated flows of analysis in the actual research were more recursive and iterative than prose can capture. This analytical workflow extended over many separate sessions across several months. The biomedical specialist conducted it in parallel with work on other research projects. In the last sub-section of Results, we present our mapping of user-centered visualization requirements for cognitive tasks in the targeted mini-flow. 

### 4.1.Objective 1: Uncover structural relationships suggesting disease-related influences

To explore possible genotype-level profiles of renal sub-diseases, the biomedical specialist began by reducing the 12,000 genes obtained through microarray analysis to 4,500 genes. To do so, he compared expression values of the measured genes for each disease patient with healthy controls, and chose only those that changed more than the estimated background. Using the selected genes for each of the 250 disease patients, the biomedical specialist then created protein-protein interaction networks for each patient. In order to find structural patterns in groups of networks constructed from his dataset, the biomedical specialist used a bioinformatics tool with capabilities for graph matching, merging, clustering, and visual analytics.

With this tool, he first ran an estimated graph matching algorithm to merge patient networks based on overlaps. The output of the algorithm was one large network. He then used a Markov Cluster (MCL) algorithm to define clusters in this large network. He repeatedly ran both these algorithms, first to assure that they were working properly and then to arrive at output with clean enough boundaries. Parameter settings determined the tightness of the clusters. During this cycling the biomedical specialist became increasingly familiar with different groupings produced by distinct parameter settings in the algorithms. When satisfied with the output and parameter settings, he had identified three clusters of interest, each with a relatively small subset of patients. The biomedical specialist disregarded other clusters from the output, all of which had fewer than three patients.

**Figure 2.**
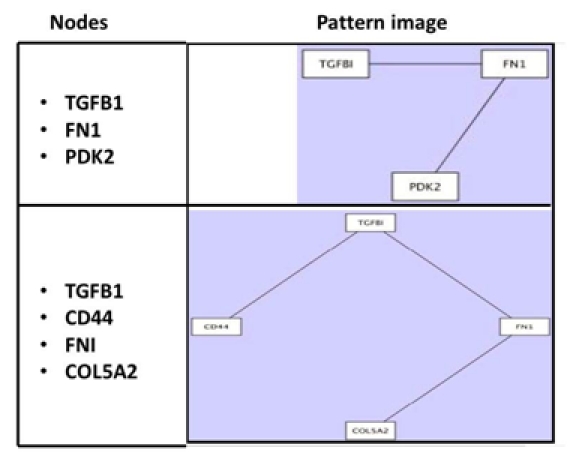
Output on subnetwork patterns for Cluster 3.

Next the biomedical specialist ran a third algorithm on each of the three clusters. This algorithm identified salient subnetworks in each of the patient networks that were included in a given cluster, as exemplified in Figure 2. The specialist also opened a heat map view of each cluster. His aim was to discern, if possible, subnetwork patterns in different patient networks within a cluster and across clusters. If he could find certain patterns characterizing most patients in a cluster the patterns might have biological meaning. Correspondingly, if some patterns were unique to only one cluster this uniqueness might signal that something related to disease was happening in this cluster.

**Figure 3.**
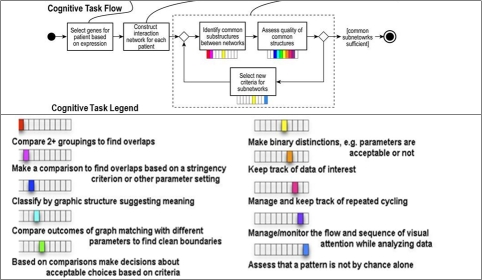
Workflow diagram of objective 1: Uncover structural relationships suggesting influences. Each box in the flow diagram is a component of core knowledge work. The bars underneath are the cognitive tasks performed for each component (color-coded). The legend shows the cognitive task related to each color and position coding.

At this point the biomedical specialist’s visual analysis mostly relied on eyeballing the heat maps and cross-referencing them with the displays of subnetwork patterns (in Figure 2). Through this informal analysis, he discerned that some subnetwork patterns in Cluster 3 were possibly distinct to this cluster; but he could not manipulate the views enough to closely compare. He nonetheless had accumulated and interpreted enough evidence combined with his structural expertise to arrive at his first milestone inference. He inferred that the clustering parameters were meaningful because they resulted in homogenous and distinct clusters. Moreover, from the clusters, he inferred that Cluster 3 especially might provide insight into sub-disease molecular traits. A flow diagram of this analytical objective is presented in Figure 3. 

The biomedical specialist was not yet done. He still needed to verify the clustering and to take additional analytical perspectives, both moves necessary for drawing defensible insights. Therefore, he shifted to a functional perspective and to his second analytical objective.

### 4.2. Objective 2: Uncover functional relationships to complement structural relationships

Here the biomedical specialist mixed a good amount of verification with interpretation. He functionally analyzed gene lists via molecular interaction networks for meaningful inferences; but he also used this functional perspective to determine if the clustering might need to be improved – and even if the prior methodology was sound. This check was essential. Methods that the biomedical specialist had used during the previous objective had a good deal of uncertainty attached to them. For example, the algorithm parameter settings that he had chosen were not based on any standard aside from his own experiential expertise. The relationships he now investigated – i.e. functional annotations and protein-protein interactions – were based on curation and data mining. These standards were also biased, serving as a check, not an assurance. 

To uncover relationships that might be functionally interesting the biomedical specialist started by identifying overrepresented annotations for genes in his dataset. For genes from each cluster, he used a tool to run enrichment analysis and visually examined groupings of annotations that were highly significant (e.g. p ≤ 10 ^-10^). He found that enriched terms for various types of inflammation response were unique to Cluster 3. His interest in this cluster grew. Cluster 2 was interesting, too. It suggested strong functional connections to microRNAs. 

Taking a related but different tack, the biomedical specialist then generated three gene lists composed, respectively, of the genes in the subnetwork patterns in each cluster. Our workflow scenario followed him only through the analysis of one of these gene lists – the list for Cluster 3. He read this list into a popular network visualization tool, Cytoscape, and took advantage analytically of several of its plug-ins (www.cytoscape.org). He sought to answer such questions as: What similarities and complementary relationships characterize molecular interactions in this cluster? What groupings of associated gene products suggest interesting indirect relationships or a liaison between pathways? 

**Figure 4. Small groupings within the network.**
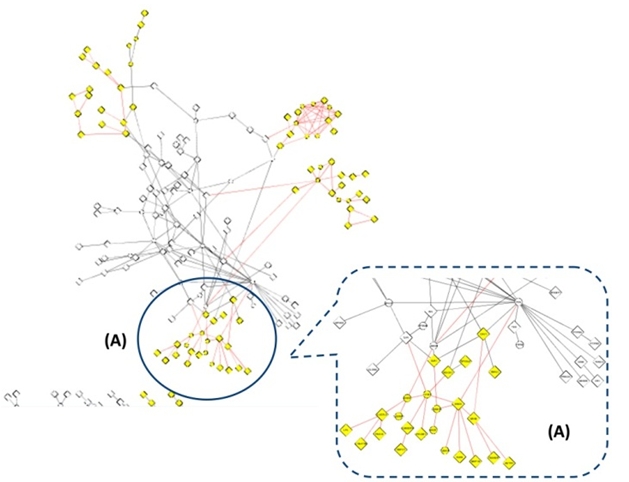
Interactors within the gene list for  Cluster 3.Possible groupings as entry points are colored in yellow. The biomedical specialist chooses to start with (A).

The network created from the Cluster 3 gene list displayed the protein-protein interactions between just the genes on the list, not first neighbors (see Figure 4). The protein-protein interaction graph and the data and functionality accessed through plug-ins let the biomedical specialist analyze traits of gene products (nodes) and traits and strengths of interactions (edges). Strengths were based on amounts of evidence, as integrated from multiple biomedical databases. As mentioned, the biomedical specialist conducted gene enrichment analysis earlier in a different tool. He chose not to use a plug-in in Cytoscape for this purpose because the workings of the plug-in algorithm were not transparent to him. Also, he had used the other tool numerous times, and felt confident and comfortable with it. The biomedical specialist, consequently, had to read enrichment results from the other tool into this visualization application. The visualization application then automatically annotated the gene products in the network with the enrichment data. In this cluster, only one term was enriched, most likely due to the stringent p-value cut-off.

The displayed network was manageable and suggested several good entry points. For the biomedical specialist a good entry point consisted of a small grouping within the whole that he could isolate and examine. He started with the grouping labeled **(A)** in Figure 4. When he finished he repeated the same flow of analysis with other small groupings (those highlighted in Figure 4). Grouping **(A)** was interesting because of its hubs, y-stars (indirect relationships) and links to the denser cluster. In other analyses the biomedical specialist had found that these topological structures often suggested biological meanings. Focusing on grouping **(A)** the biomedical specialist spent a good amount of time iteratively exploring the subnetwork and its data to increase his familiarity. Recursively, he classified relationships based on shared annotations. He used color coding to bring in the traits, first looking at only one trait at a time and then progressively looking at two or three shared traits together. He did not examine more than three shared traits at once because the cognitive load was too great. He manually grouped similarities as he went along. At times, he color coded by annotations provided by the tool, and at other times he color coded by only the overrepresented annotation in this cluster, which was MHC-II inflammation response. The biomedical specialist cycled through many traits and arrangements. As a heuristic strategy the cycling allowed him to better remember relevant attributes and relationships and to become more confident if they occurred repeatedly. 

As the biomedical specialist began to uncover some potentially interesting relationships of similar and complementary traits, he wanted to construct them into his own groupings of nodes based on two traits but could not do so automatically in the program. Instead he had to arrange nodes manually, an extremely time-consuming endeavor. These manual arrangements involved using many other program operations in conjunction with moving nodes and edges, e.g. selection, filtering, and zooming. The biomedical specialist often interacted with a dynamically linked data panel to sort, read, and select various details about nodes of interest. His cognitive load grew high, and as he progressed he often saved and bookmarked views as best he could. To better direct his selective analytical attention, he color coded some similarity groupings of genes that seemed particularly interesting for later recall.

For the manually grouped relationships that were interesting in terms of pathways, enrichment, and other specific biological processes, he examined their edge traits. Edge details showed the amount and type of evidence supporting each pairwise interaction. The more sources of evidence the biomedical specialist saw for an interaction, the greater his confidence became. For some edge details he could infer types of interactions, as well. For example, if an edge indicated that evidence of an interaction came from yeast 2 hybrid methods the biomedical specialist knew it was a physical interaction, albeit with a high chance of being a false positive. Biological information behind edge details unfortunately was incomplete; and the plug-ins did not actually do any semantic categorizing of types of interactions. Later, in making a transition to more contextually grounded explanations, the specialist would require more semantically precise details about interaction types.  

**Figure 5. Workflow diagram for objective 2: Uncover functional relationships.**
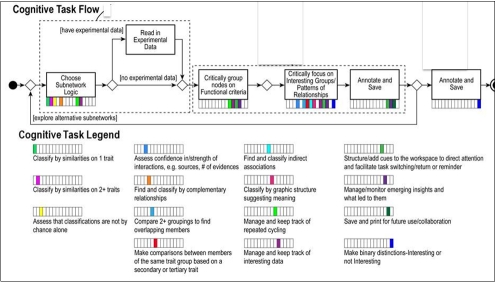
Each box in the flow diagram is a component of core knowledge work. The bars underneath are the cognitive tasks performed for each component (color-coded). Single cognitive tasks often repeat across components; yet no component has the same combination.

When the biomedical specialist had completed an analysis of grouping (A) he turned to the next small grouping in the network (another one of the highlighted groupings in Figure 4). He continued this analysis – small group by small group - until he knew enough to be able to perform the same sort of analysis with a larger network composed of the first neighbors of select genes. Based on analyzing these smaller subnetworks first he later could manageably tackle a larger network. He could eliminate from the larger network many irrelevant annotation-based relationships found in the subnetworks. When these rounds of analysis on Cluster 3 were done, the biomedical specialist performed the same workflow for the other two clusters, which are not presented here. This workflow is presented in Figure 5. 

By the end of the second analytical objective the biomedical specialist had uncovered evidence and warrants that advanced his earlier inference about Cluster 3. Thanks to functional enrichment and network analysis, he identified specific genes and gene interactions in Cluster 3 that were tied to different responses to inflammation. MCH-II inflammation proved to be important in relation to other traits and interactions in the Cluster 3 network. Moreover, MCH-II inflammation was only an enriched term for Cluster 3. The biomedical specialist inferred that associations connected to MCH-II inflammation were credible and could be relevant to mechanisms of disease variations. A relationship between inflammation and epigenetics was particularly promising for further investigation, and it was novel. At the end of this analytical objective, the biomedical specialist also had findings on Cluster 2 that raised new questions about the workings of transcription events. He now turned to collaborations with his clinical colleagues. Their expertise would help direct his further analysis.

### 4.3. Offline: Gain knowledge by consulting clinical colleaguesand reading literature

Collaborations took place mostly offline. The biomedical specialist and his clinical research colleagues examined the saved views and assessed notes that the biomedical specialist had written on them. The collaborators re-composed, synthesized and interpreted across biological scales – now reaching into clinical data, quantitative traits for phenotypes, and known insights from the field of renal disease. They found some concordance between molecular and clinical phenotypes but also variances. For example, for Cluster 2, they found cell lines treated with hydrocortisone were linked with the transcription annotations. The biomedical specialist also searched relevant research literature. This evolving analysis of structures, functions, and now biological context gave unity to his efforts to uncover possible mechanisms of sub-diseases. 

### 4.4. Objective 3: Gain insight into biological events from expression values in networks

With this unified frame of reference, the specialist embarked on his third online objective. He valued the explanatory power of overlaying expression data on richly annotated molecular interaction networks, and he hoped this strategy would now help him infer how genes were wired into each cluster-based network. Focusing closely on expression data at this point the specialist’s search for meaningful interactions widened but the risk of faulty interpretation also increased. Because of this trade-off he was particularly cautious about trusting the data, his interpretations, and inferences. 

He started with Cluster 3 again and began to examine expression level differences across the 12 patients who made up the cluster. He set up the tool’s animation capability with 12 stop points, one for each patient. He then spent a good amount of time setting up the protein-protein interaction network for Cluster 3 in ways that would help him discern patterns as the expression values animated. He sized the nodes by expression value, and he manually arranged nodes to maximize the chance of seeing meaningful animation views. He clustered expression values and grouped together genes with similar or complementary traits. 

**Figure 6. Workflow diagram for objective 3: Gain insights into biological events from expression values in networks.**
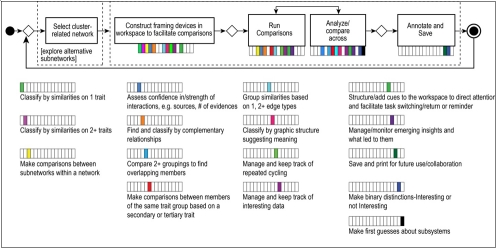
Each box in the flow diagram is a component of core knowledge work. The bars underneath are the cognitive tasks performed for each component (color-coded). Single cognitive tasks often repeat across components; yet no component has the same combination.

When he finished arranging the view strategically, he clicked on each stop point, and the expression values for each patient network highlighted. Along with the highlighting, the biomedical specialist mentally registered other properties associated with the lit-up nodes – e.g. the node attributes, interactors, and configurations. As best he could, he made mental notes at each stop point about genes that might be contributing to abnormalities, and about similarities and differences that might be important across stop points. He ran the animation numerous times to help make these interpretations. These mental comparisons and spatial transformations severely taxed his visual memory. The workflow is shown in Figure 6.

To relieve the cognitive load and to make a closer, side-by-side comparison, he captured each view and saved it. After studying the captured views more closely and finding patterns, the biomedical specialist arrived at his third milestone inference for hypothesizing. He inferred that certain patterns among up and down regulated genes and their interactors in Cluster 3 might be influencing a novel inflammatory response tied to epigenetics. 

The biomedical specialist now consulted further with his clinical research colleagues and read more deeply in the literature. This marked the end of the workflow that this case portrays. After consulting with colleagues and the literature, the biomedical specialist would begin a new workflow, one that comprised deeper causal and conditional analyses of biological events. 

### 4.5. Mapping cognitive tasks to user requirements 

With the workflow of this case concluded we now examine the ways in which the representations of it coupled with one more workflow representation can help guide prototype development of a visualization tool. The additional representation is our mapping of user-centered requirements to the cognitive tasks in the workflow. To create the mapping, we selected a mini-flow pertaining to the second analytical objective. The mini-flow only covered classifying gene products functionally by two or more similar traits (refer back to Figure 3). It omitted other aspects of the second objective, such as classifying by complementary traits, patterns, or making comparisons. In practice all these reasoning modes were interwoven but for proof-of feasibility - our scope - we sought success with a small subset first, signaling a go- ahead to scale up in further research. 

**Figure 7. Tasks to requirements:**
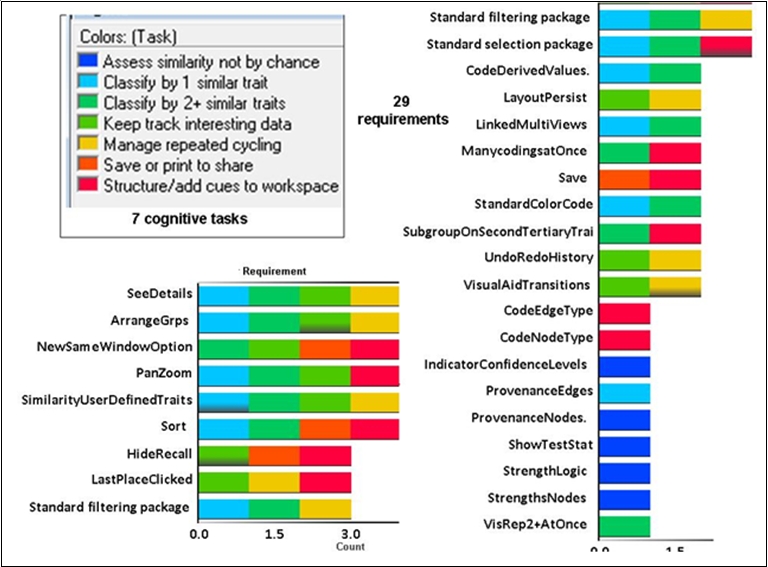
For the mini-flow of selecting and grouping genes based on two or more similar traits seven cognitive tasks occurred. The cognitive tasks are color coded as specified in the top left legend. This figure shows the seven tasks mapped to requirements necessary for supporting them (the labeled bars in the figure). Striped colors in a requirement bar indicate the different cognitive tasks that the requirement addresses. Some requirements support as many as four tasks; some support only one. Bar length in the requirements bar graph indicates the number of tasks mapped to a requirement. If the graphing and color coding were reversed and the bar charts were to show cognitive tasks, color coded by requirements, the task bars would show a similar many-to-many mapping.

The mini-flow involved seven cognitive tasks (specified in Table 2), which combined in various ways for actual analysis. Twenty-nine user-centered visualization requirements (Figure 7) mapped to these cognitive tasks. Supplemental Material 2 gives details on these requirements. In this mapping the requirements related to selecting, color coding and filtering are designated as “standard packages.” A standard package unifies numerous requirements that the research literature shows to be well-established for these visualization techniques in complex analysis. 

**Table 2.**
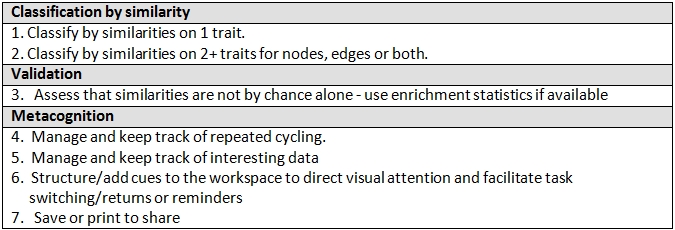
Subset of cognitive tasks for mapping to user requirements

As Figure 7 shows, even a small subset of seven cognitive tasks for this mini-flow demanded complicated support. For example, a good number of the task-to-requirement mappings involved many-to-many relationships. Figure 7 visually shows the mappings in a way that avoids the multi-edge crossings of many-to-many relationships. In many instances, one requirement applied to many tasks; and a single task had more than one requirement. Additionally, a single task often recurred in various components of core knowledge work, but combinations for each component were distinct. 

Figure 7. Tasks to requirements. For the mini-flow of selecting and grouping genes based on two or more similar traits seven cognitive tasks occurred. The cognitive tasks are color coded as specified in the top left legend. This figure shows the seven tasks mapped to requirements necessary for supporting them (the labeled bars in the figure). Striped colors in a requirement bar indicate the different cognitive tasks that the requirement addresses. Some requirements support as many as four tasks; some support only one. Bar length in the requirements bar graph indicates the number of tasks mapped to a requirement. If the graphing and color coding were reversed and the bar charts were to show cognitive tasks, color coded by requirements, the task bars would show a similar many-to-many mapping. 

### 4.6. Implementing a prototype from a trial mapping

**Figure 8. Prototype mock-up.**
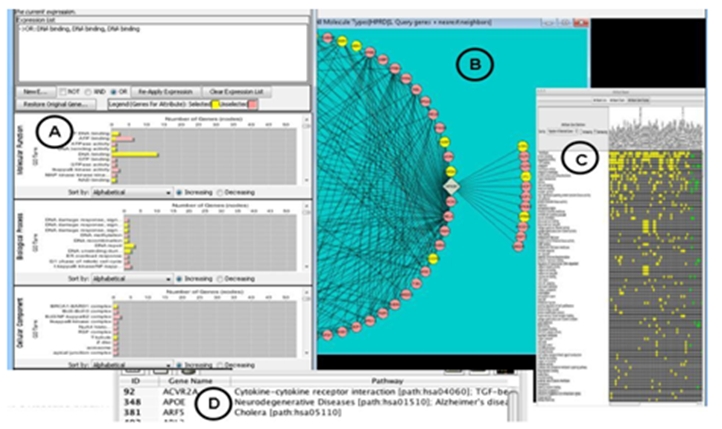
Bar charts of attributes are shown in (A) - in this case classes of GO terms. These bar charts detail all attributes associated with the gene products displayed in the network view, shown in (B). In (A), bar length indicates the number of times an attribute occurs as a trait of the gene products in the network. When a scientist selects 2+ attributes from the bar chart, the associated gene products in the network (B) and matrix (C) are dynamically highlighted. Additional attributes in (A) that co-annotate the highlighted proteins also are dynamically highlighted. The matrix cells in (C) show the attributes (rows) that annotate each gene (columns).   By interacting with the matrix, a user can filter to only certain attributes, and the views will show only the genes having those attributes of interest. Results dynamically update across the graph, bar charts, and data table (D). A user can set various visual codings to indicate traits of most interest  and can manually group gene products in the network that  share two or more traits. The user can view the strength of interactions in potentially interesting groupings by referring to the details on edges in the data table. These four dynamically linked views should foster a fluent and complete flow for the objective of selecting and grouping by 2+ similarities.

To develop a prototype informed by the tasks-to-requirements mapping, usage scenario, flow diagrams, and task taxonomy, we worked with a senior developer. The aim was to create a visualization prototype that would satisfy the bundles of requirements related to selecting and grouping data based on multi-dimensional similarity. The senior developer had little trouble translating the requirements into language that other developers could follow, signaling that our levels of granularity in tasks and requirements were effective. He implemented the requirements in a prototype Cytoscape plug-in, the interfaces of which are shown and explained in Figure 8. 

Overall, this proof of feasibility suggested that fit-to-purpose visualizations for individuals’ actual approaches to this workflow could be achieved through the interrelated representations we constructed and present in this article. 

## 5. Discussion

Our workflow research takes a holistic, user-centered perspective in order to uncover the interconnections between a biomedical specialist’s analytical objectives, the core components of knowledge work he performs to achieve each objective, and the cognitive tasks he performs in each component. Our study implicitly shows that a great deal of expert knowledge and many heuristic strategies underlie this cognitively-oriented workflow. For example, the biomedical specialist’s expertise in structural analysis guided his interpretations throughout the workflow, and especially during his first objective. This expertise is tacit and includes, for example, knowing: (1) What counts as an acceptable structure; (2) what methods to use to generate acceptable structures, e.g. parameter settings and diverse perspectives; (3) when and how to cross-reference different types of structures; (4) when and how to comparatively analyze overlapping structures and what can and cannot be compared (especially challenging in a large network); and (5) what limits to set on the number of dimensions to analyze together so that visual analytics is manageable. Similarly, heuristic strategies tacitly informed many of biomedical specialist’s cognitive tasks. For example, he repeatedly cycled through the same data display to internalize a familiarity with the data, to reduce cognitive load, and to maintain coherence (30). As other heuristic strategies, he limited the amount of displayed information at any one time to avoid cognitive overload and distraction, and marked emerging relationships of interest with cues. As detailed later, cues helped in maintaining coherence and in keeping track of inquiry paths and progress. 

Our case and representations concentrate on reasoning and action at an application level. At this level, they reveal four interrelated propositions with implications for the development of visualization tools. Each proposition is discussed below, and we end with implications for tools. 

### 5.1. Higher order reasoning is multi-modal, compositional, and inseparable from a scientist’s active manipulation of knowledge representations. 

Our findings reinforce prior research in science studies that stresses the compositional nature of higher order thinking-and-doing for complex analysis (7). In our case, the biomedical specialist mixed many modes of reasoning – classification, comparison, validation, and metacognition. He did not mix in predictive mental modeling or story-telling. These modes probably come into play in the next, more in-depth workflow. For the modes of reasoning that he applied, he enacted numerous facets for each mode. He also combined multi-faceted cognitive tasks distinctively for each goal-driven component of core knowledge work. Our task taxonomy identifies 35 faceted cognitive tasks that recur in this workflow. For example, when comparing data, the biomedical specialist enacted such facets as “Compare two or more groupings to find overlapping members” and “Compare outcomes/graphs from overlap analysis based on different parameter settings.” For classification, he grouped genes by two or more similar attributes (e.g. GO annotations) and by complementary attributes (e.g. pathways and GO annotations). By capturing these multi-faceted cognitive tasks, our results provide greater cognitive specificity than is found in other cognitive models of scientists’ discovery-based reasoning in the current literature.

Moreover, the case demonstrates that at an application level higher order reasoning is inseparable from manipulating knowledge representations to draw inferences. This finding reinforces arguments from requirements engineering. These arguments claim that the degrees of freedom that an electronic workspace gives an analyst for adaptively manipulating views and data for discovery purposes strongly influence the success of the analyst’s investigation (31).

### 5.2. A cognitive task has an analytical function only in relation to a unified set of tasks.

Discrete cognitive tasks or taxonomies of tasks cannot reveal the situated analytical functions that a scientist’s cognitive tasks serve in the flow of a complex analysis. Rather function is tied to combination (32). At an application level our representations reveal combinations of cognitive tasks associated with goal-driven components of core knowledge work. These combinations are depicted in our workflow diagrams and in the tasks-to-requirements mapping in Figure 7. Because our findings show distinct combinations of cognitive tasks for each goal-driven component of knowledge work they reduce what would otherwise be a daunting number of possible combinations of cognitive tasks for tools to support. The combinations that advance specific objectives can help developers understand what to target for tool support. 

In tool development for the complex analysis presented in our case, supporting analytical functions of cognitive tasks as they combine with other cognitive tasks is challenging. Often a single visualization technique will have to support several different analytical functions for a given workflow objective. For example, the biomedical specialist used color-coding to classify similarly annotated genes; he also used color-coding metacognitively to cue salient relationships that he wanted to recall. In the same vein, his manual grouping of nodes and edges served several functions. It helped him classify logically; and it also helped him metacognitively cue protein interactions that he deemed important, and assess the completeness of the data. Put simply, numerous visualization features and techniques, when tied to the analytical functions of cognitive tasks, must be robust, i.e. applicable to a wide range of functions. Therefore, they have to be developed with a good deal of flexibility and adaptability. This issue is discussed in more detail more below. 

### 5.3. Knowledge work components focus on negotiating meaning, not just read-offs.

Our case reveals that a large part of the biomedical specialist’s combined cognitive tasks were functionally aimed at negotiating meaning. Negotiating meaning included but far exceeded reading off data from a display. Cumulatively, the biomedical specialist negotiated meaning by using built-in algorithms and by actively interrogating the workspace displays and affordances. He sought to uncover non-obvious relationships and diverse perspectives relevant to each analytical objective. Additionally, he sought to synthesize emerging knowledge across objectives. Within and across the flow of each analytical objective, he moved through different modes of logical analysis (classification and comparison); reconciled uncertainty (validation); stayed oriented, and monitored and managed progress (metacognition). He drew relevant meanings from various structural, functional and regulatory perspectives for each separate analysis of the three patient clusters. Moreover, he iteratively analyzed many subnetworks within each cluster and overlaid regulatory patterns on each cluster’s network. In summary, recalling Trickett and Trafton’s work on spatial transformations for deep insights and inferred meanings, the biomedical specialist spatially arranged, classified and compared externally displayed knowledge representations to construct new knowledge relevant to his research questions. He did so in almost every slice of time (i.e. in a single component of core knowledge work), and cumulatively across time (across knowledge work components both within an analytical objective and across objectives).

Progressively these manipulations led the biomedical specialist to develop emerging mental models of credible and plausible genes and relationships possibly influencing renal disease. In other words, the processes of negotiating meaning, filling out mental models, and interacting with workspace affordances dynamically shaped each other. Unfortunately in bioinformatics, our understanding about how to facilitate and enhance this dynamic interplay is a relatively unexplored area. From an information visualization perspective, Liu and Stasko offer insight. They explain mental models in ways they hope can help developers understand and design for the external-internal dynamics of sensemaking (33). Mental models, Liu and Stasko expound, are internal explanatory representations relevant to an analyst’s research question. An analyst progressively constructs them by interacting with external displays of data, especially with displays of structural and functional relationships. As the case we present above shows, the biomedical specialist paid attention externally to these structural, functional, and regulatory relationships and mentally constructed plausible models of potential influences. 

From a science studies perspective, Neressian explains mental models further. She explains that in progressively developing problem-related mental models scientists strive to understand one representation of knowledge – externally or internally - in terms of another (34). In fact, scientists typically formulate several intermediary representations of knowledge from diverse data and sources to order to move from a starting point of understanding to an inference. In the renal disease case, these intermediary representations took the shape of the multiple perspectives that the biomedical specialist used to conduct his three online objectives. Explanations of mental modeling in the research literature typically stress analysts’ logical processes. Results from our study reveal that metacognitive processes are equally important to developing inferences and the mental modeling associated with them, as we now explain. 

### 5.4. Metacognitive tasks are prominent in generating inferences.

Our mapping of cognitive tasks to user-centered requirements for the mini-flow used in our prototyping shows that in our case metacognitive reasoning-and-action came into play as frequently as logical reasoning, i.e. as classification. For example, the biomedical specialist kept track of intermediary representations/diverse perspectives relevant to inferences he drew, making metacognition an intrinsic part of mental modeling. Empirical research in science studies reinforces this finding. Researchers have shown that continuous metacognitive thinking occurs, for example, in scientists’ “explicit articulation and reflective refinement of methods, reasoning strategies, and representational issues” (34, 7). 

Additionally, metacognitive thinking is central to maintaining a coherent cumulative flow of analytical inference within and across analytical objectives. Our case makes clear that inferences are not self-contained actions, and tool developers should not presume to support them as such. Rather they are the product of a progressive flow of cognitive tasks and objectives. Metacognition helps scientists keep track of knowledge and inquiry paths in this flow. As usability tests show, positive user experiences depend on uninterrupted flows in higher order reasoning (35). Unfortunately, as researchers have noted, the greatest insufficiency in applications today is support for streams of goal-driven, coherent interactions (36).

One of the biomedical specialist’s prime ways of making his workflow coherent was to repeat earlier moves and strategies but with new content and structures. He also relied on comparing past and present views. For such recursive analyses and comparisons he needed to maintain a keen sense of his activity history and relevant outcomes – i.e. what we call activity provenance.

The biomedical specialist also achieved coherence by leaving himself cues, dozens of them to direct his visual and intellectual attention. This strategy is common to expert problem solving (37). Cognitively, cues “jig” the informational environment to decrease its variability: “The more completely prepared an environment is for one’s problem and purpose the easier it is to accomplish one’s task” (38, 37). For example, cues – whether set automatically or manually – can signal shifts in task paths, changes in the state of the problem space, confidence levels, or missing information. Cues also may indicate important points, views, or interactions to remember or recall. They may indicate structural or semantic patterns relevant to inferences; competing groupings for judging relevance; or items or views to share with collaborators. Cues enable selective attention. Dumais and Czerwinski highlight the importance of selective attention, arguing that for exploratory analysis, “attention is a critical resource and is what matters if [a software] system is to be usable.” (39,1362). Basically, cues direct attention selectively to help domain experts stay oriented; formulate what they know and still need to know; and determine the next moves, strategies, and analytical paths that will be coherent with preceding paths. 

The biomedical specialist needed this orientation, especially when he ran animations of expression data. Without being able to set cues he had to depend on repeated runs and unreliable visual memory. Help from a workspace for cuing as well as for seeing action trails is vital for advancing a scientist’s internal problem solving for inferences and for sharing with collaborators. 

### 5.5. Implications for visualization development 

As the discussion above shows, the targeted workflow presents many demands that visualization workspaces must support effectively and efficiently. We have organized these implications for visualization support into three types relevant to the targeted workflow: (1) “Heavy lifting;” (2) important but underdeveloped support for finding and trusting meaningful relationships; and (3) metacognitive structuring. For each area of support, we first propose priorities in terms of development approaches as bulleted items points and then explain the need for acting on these priorities. 

*1. Heavy-lifting.* The need for visualization tools to improve what we call heavy-lifting is implied by our first two propositions – namely, that cognitive tasks are compositional and that analytical functions result from combined – not isolated – cognitive tasks. The development priorities that we propose related to these propositions include:

Design and develop the following features with a great deal of flexibility and adaptability based on the analytical functions that they must support in flows of cognitive tasks and components of knowledge: Color coding; techniques for arrangement (including layout, sorting, subgroupings on two and three traits); filtering; providing multiple dynamically linked views that can foster different perspectives; and searching and selecting.
Design and develop bundles of features that adequately support single, application-level cognitive tasks.

In regard to the first priority, the case shows, as previously mentioned, that a single visualization technique often supports two or more integrated cognitive tasks, and the tasks often serve different functions in diverse components of core knowledge work. In the biomedical specialist’s workflow color coding and layout /arrangement took on a large “support burden” (heavy-lifting) to serve these different analytical functions. Thus these visualization techniques need to be designed with a great deal of built-in flexibility and adaptability. The other features mentioned in the first bullet point are gleaned from the scenario and the mapping of the mini-workflow. Supplemental Material 2 describe relevant packages of capabilities for many of the priority features.

We believe that mappings like ours can help developers identify features that need to be flexible by showing which requirements come into play frequently, given recurrent cognitive tasks in different components of core knowledge work. After developers have created preliminary prototypes, our scenarios and workflow diagrams can help them get a reality check on the choices they have made about flexibility and adaptability. The scenarios and workflow diagrams can show if the choices accommodate the distinct combinations and functions of cognitive tasks in a biomedical specialist’s actual analysis. For example, if development choices automate numerous moves for efficiency’s sake, the scenario will show that moves such as repeated cycling should not be “automated away.” A scientist needs this recycling to build knowledge, familiarity, and confidence. 

The second bulleted priority above deals with the fact that many single cognitive tasks – outside their relation to others – need support that involves a “bundle of requirements” at once. In the mini-flow we mapped, for example, the biomedical specialist needed nine requirements in place to perform the metacognitive task of managing repeated cycling. (Readers can reference these nine requirements in Figure 7 by looking at the bars (requirements) with a yellow stripe.) Traditional feature lists do not capture this need for multiple requirements to work together smoothly as a bundle. Neither the functional nor bundled orientation that we propose here is common in bioinformatics development of visualization tools. To foster this approach this study offers a first pass at defining relevant combinations and bundles. The importance of further research into what these bundles are cannot be overemphasized.

*2. Important but underdeveloped support for negotiating meaningful relationships.* In visualization tools support is often underdeveloped for a biomedical specialist’s central activity of negotiating meaning for his or her research questions. For our targeted research question and workflow we propose the following top priorities for user support:

Dynamically linked views that facilitate making comparisons and facilitate marking important aspects within and across views
Capabilities for making comparisons through network overlays
Semantic content on types of interactions; and ways to analyze details on 2+ edges in user-defined groups of protein-protein interactions

As the case shows, a good amount of the biomedical specialist’s negotiating of meaning involved grouping - e.g. by similarity, complementarity, structure, overlap, and user defined sets of traits. Many of these grouping activities need to be addressed through the development of the more flexible and adaptable support mentioned previously. The priorities in this sub-section present additional support.

To negotiate meaning, the biomedical specialist depended strongly on making diverse comparisons, which included mental comparisons. For example, he kept many comparisons in visual memory as he animated expression patterns overlaid on the network. The biomedical specialist also consistently validated and frequently returned to prior parts of the analytical flow for checks while negotiating meaning. When he did, his cognitive load was high, partly because necessary tool support was underdeveloped. Finally, after a certain amount of narrowing down to interactions of interest, the biomedical specialist’s analysis of edges became uppermost. It was essential for him to examine and compare the types and strengths of relationships connecting groups of genes that were grouped together by several meaningful attributes. Interrelating details on more than two edges was mentally taxing. The priorities above target visualization techniques and content that can alleviate this cognitive load.

*3. Metacognitive structuring*. The need to support metacognition harks back to the earlier discussion about the importance of action trails (activity provenance) and a scientist’s need to set cues in the workflow. For this focus, our proposed priorities for visualization development are:

Functionality for tracking, querying, and re-playing activity
Undo and bookmarking capabilities
Functionality for scientists to actively “seed” the workspace with various subjective cues

Our case suggests that in developing activity trails to support metacognitive thinking, programs need to automatically chunk captured activity and present it to scientists at an application level – i.e., at the level at which biomedical specialists conceptualize their analytical actions. Our components of knowledge work and constituent cognitive tasks can help to guide this chunking. Additionally, cuing needs to be sensitive to complicated instances, for example the biomedical specialist’s flow of animation-based comparisons. Liu and Stasko underscore the difficulty a scientist faces in keeping track of such animated comparisons (40). As they note, “It is non-trivial to identify the combination of values that actually lead to [an] abnormality;” and to keep track of these values visual cues are essential (40, 1027). 

## 6. Conclusions

This study focuses on a workflow that often gives scientists difficulty due to insufficient software support. We uncover the human constraints that need to be considered if network visualization tools are to satisfy biomedical specialists’ actual ways of knowing and reasoning for this class of analysis. We represent these constraints as analytical objectives and link these objectives to their associated core components of knowledge work and the combinations of cognitive tasks enacted for each component. Our study is an initial step in putting together patterns of actual analysis at an application level. When such patterns are more fully established they may help developers intelligently pre-define and build in greater flexibility, improve support for negotiating meaning, and better facilitate metacognitive thinking.

Our study also suggests that developing robust tools will be an incremental process. Our prototyping efforts intimate that by mapping just a mini-flow developers can construct prototypes that fit scientists’ goal-driven cognitive tasks. We envision that by conducting user performance evaluations on such prototypes development teams would uncover and implement further improvements. Visualizations thus would steadily improve for the aspects of a workflow targeted by a prototype. Scientists would then be able to better clarify and articulate their needs for the parts of the workflow that are not yet supported. These specified needs would lead to additional mappings and continued improvements for usefulness. Our approach should help biomedical computing break out of the current mode in which technology specialists build applications for bioinformatics-oriented research rather than for the exploratory translational research that collaborating biomedical specialists want to perform for context-rich research problems. Without adequate tools, investigators often conduct their work across too many platforms without coherence. 

The mapping and other representations that we have created hold promise for visualization development in other ways, as well. They could help developers set priorities and select from the start programming tools that best support scientists’ goal-driven cognitive tasks. They also could help to reduce costs by efficiently guiding development for usefulness from inception on. Additional cases of cognitive task analysis, field studies, mappings, and prototyping are needed for greater understanding and generalizability. We believe that additional research will uncover discernible patterns characterizing the fluent performance of this discovery-driven workflow. Developing tools for these patterns is a crucial frontier to cross. Fit-to-pattern visualization tools are necessary to assure that a broad base of biomedical specialists – and not just early adapters - can engage in effective and efficient translational research.

## Acknowledgments: 

This study was supported in part by the following grants from the National Institute of Health: U54 DA021519 , P30 DK081943, and RO1LM00981201A2. Correspondingly, it was performed in part under the framework of the National Center for Integrative Bioinformatics (NCIBI) and the Applied Systems Biology Core of the O'Brien Kidney Research Center at the University of Michigan. We thank Fan Meng and An Blakeslee for helpful comments on earlier drafts. We are grateful to Terry Weymouth for developing the visualization prototype from the findings of our cognitive task analysis. We also are indebted to our graphic artist colleague, Paul Trombley, for assistance in creating the workflow diagrams. 

## Authors’ contributions.

BM conceived of and led the research project and participated in all phases of the cognitive task analysis, data analysis, and construction of representations. She helped to guide the prototype development, and she collaboratively wrote the manuscript with FE. FE was the subject of the cognitive task analysis. He gave feedback on findings and participated in numerous cycles of constructing and re-constructing the workflow representations. He helped guide the prototype development and create images of it for presentation. BK participated in the first phase of the cognitive task analysis and played a major role in rendering the flow diagrams. He also reviewed drafts of the manuscript and the representations. MK provided biomedical expertise and collaborative consultation related to the case. 

## Supplementary Material

Supplemental Material 1: Cognitive tasks for the targed workflow organized by mode of reasoning - Research Instrument Click here for file

Supplemental Material 2: Requirements for selecting and grouping by 2+ similarities - Research Instrument
Click here for file
