# Supplementary material for: A cognitive task analysis of a visual analytic workflow: Exploring molecular interaction networks in systems biology
Source: J Biomed Discov Collab. 2011 Mar 21;6:1–33. doi: 10.5210/disco.v6i0.3410 (PMC3090070; doi:10.5210/disco.v6i0.3410)
Supplement: Supplemental Material 1: — Cognitive tasks for the targed workflow organized by mode of reasoning - Research Instrument [file Jbiomeddiscovcollab-06-e01-s001.pdf]

## Supplemental Material 1:

## Cognitive Tasks for the Targeted Workflow Organized by Mode of Reasoning

NOTE: Validation is embedded in each reasoning mode

### 1. Classification: By similarity

| | |
|---|---|
| a | Make binary distinctions – e.g. judge known vs unknown data, interesting or not, best choice vs contending options, significant or not |
| b | Classify by 1 similar trait or metadata or value/value range shared by members. Involves characterizing each item by a trait and seeing both the shared trait and the members. |
| c | Classify by 2+ similar traits or metadata or value/value range shared by members |
| d | Group similarities based on 1, or 2+ edges (types of relationships) connecting members |
| e | Assess types of and confidence in interactions (edges) by some validation criterion |
| f | Assess that similarity membership is not by chance alone/judge significance level |

### 2. Classification: By patterns [groupings within or between groupings]

| | |
|---|---|
| a | Find and classify indirect associations or interactions |
| b | Find and classify by complementary relationships (i.e. sequences or sets of links, their interaction types, and their strengths are important or indirect clusters and member gene) |
| c | Classify by graphic structure suggesting meaning, e.g. outlier, distribution, liaisons between sub-networks, motifs |
| d | Group by graph theoretic computations to suggest meanings, e.g. centrality, connectedness |
| e | Break down a large network to classify it into sub-networks by some criterion |
| f | Find and classify by temporal (sequential or looped) relationships (e.g. time series, regulatory, fusion) |
| g | Find and classify by spatial/physical context relationships |
| h | Assess that a sub-network of associations or pattern (e.g. graph theoretic structure) is not by chance alone |
| i | Assess that direct and indirect associations between groups are not by chance |
| j | Assess confidence in strengths, plausibility, and/or credibility of relationships/links |

**3. Classification: By subsystems**.

| | |
|---|---|
| a | Make preliminary inferences as available evidence and tool capabilities permit about about patterns and/or conceptual relationships among members that may warrant classifying them as a functional subsystem - e.g. first guesses about protein classes, cascading interactions, combined regulatory, pathway, and structural relationships; biological coordination processes |
| b | Assess evidence to establish confidence |

**4. Comparison**

| | |
|---|---|
| a | Compare 2+ groupings to find overlapping members/interactors - e.g. network sub-graphs that represents the associations shared by 2+networks |
| b | Make a comparison to find overlaps based on a stringency criterion or parameter |
| c | Make comparisons between members of a same-trait group based on a secondary and/or tertiary trait |
| d | Compare clinical traits and molecular functional relationships amid certain and uncertain criteria |
| e | Make comparisons between the same data in different graph layouts |
| f | Make comparisons between sub-networks of a large network derived from the same sub-dividing logic. |
| g | Make comparisons between sub-networks of a large network derived from the same sub-dividing logic. |
| h | Compare and/or synthesize different data in different graphic forms and relationships |
| i | Compare outcomes of graph matching run with different parameter settings to find views with the cleanest boundaries |
| j | Judge algorithm logic and patterns to be acceptable |
| k | Assess that sub-network divisions are not by chance alone, when possible |

**5. Metacognition**

| | |
|---|---|
| a | Manage and keep track of repeated cycling in analysis and know when to move on |
| b | Structure/add cues to workspace- to direct attentiona and facilitate task switching, recall, tangent and return |
| c | Manage and monitor emerging insights as well as dead ends and lines of thought and actions leading to them |
| d | Manage and monitor flows and sequences of visual attention while analyzing data |
| e | Keep track of data of interest |
| f | Save and/or print, projecting to collaborations and/or future uses |
