# Supplementary material for: A cognitive task analysis of a visual analytic workflow: Exploring molecular interaction networks in systems biology
Source: J Biomed Discov Collab. 2011 Mar 21;6:1–33. doi: 10.5210/disco.v6i0.3410 (PMC3090070; doi:10.5210/disco.v6i0.3410)
Supplement: Supplemental Material 2: — Requirements for selecting and grouping by 2+ similarities - Research Instrument [file Jbiomeddiscovcollab-06-e01-s002.pdf]

Supplemental Material 2: Requirements for Selecting and Grouping by 2+ Similarities

Users can:

1. Use many visual codings or other visual/Gestalt techniques to set many cues at once. That is, for multi-focus interaction, users can give prominence to a number of high priority information for seeing patterns and relationships: GO annotations, homology, pathways, reactions, interrelated layers of GO annotations across classes, overlap [Dadzie and Burger; Seo and Shneiderman; Elmqvist et al.]

2. Arrange into groups. Spatially arrange data items into groups manually or through pre-defined layouts or orderings (e.g. to see overlaps) and save the new arrangement (e.g. as a state that can be saved or returned to/bookmarked) if users specify to do so. [Seo and Shneiderman; Dradzie and Burger; Yi et al., Akers et al.]

3. Standard color and other coding package: Code many codings at once (e.g for logical analysis) [Uetz et al., 2002; Schrinivasan and van Dijk; Kang et al. (a); Thomas and Cook; Tominiski et al.; Brewer] Users can:

> code by color, luminance, size, shape, line width, line style
>
> override default or other program-set coding.
>
> color or otherwise code on attributes, numeric values, types of relationships, types of entities, time, literature metadata, user-defined range/grouping of values in a field.
>
> color or otherwise code and change the coding efficiently (1-3 steps)
>
> be assured that their codings carry over for a number of moves without defaulting to initial settings
>
> add to, subtract from, or change them as needed in minimal steps; and be able to return to prior moves with prior coding intact.
>
> show and hide legends for codings and legends update as codings change

4. Code on derived field values. Perceptually code node and edge traits on derived fields or values, including counts or types of experiments that showed a particular interaction, aggregates [Barsky et al, 2007]

5. Show/color by test statistics for clustering and other groupings, e.g. correlations, enrichment statistics, t-tests. [Seo and Shneiderman; Wong et al.; Perer and Shneiderman]

6. See indicators of confidence levels when statistical values are encoded by color, size, thickness [Uetz et al; Holloway et al.]

7. Show strengths of nodes [Seo and Shneiderman; Piringer et al.; Wong et al.]

8. Reveal the logic of this strength [Seo and Shneiderman, 2006; Piringer et al.; Wong et al.]

9. Give access to provenance information on nodes. See and link to the source databases and/or the provenance of attributes of entities [Seo and Shneiderman; Saraiya et al.]

10. Undo, redo, go back to a point in the flow/history and go home and have the designated state and view intact. [Schrinivasan and van Dijk;Piringer et al.; Akers et al.]

11. See the action trail behind saved/bookmarked views, including links to tools, data and/or author creating them.//insight provenance for collaboration// [Gotz and Zhou; Robinson]

12. Immediately see the last place they clicked in a graphic [Kang et al. (b), Robinson]

13. Visually represent relationships of similarity characterized by 2+ attributes or values at once. [Dadzie and Burger]

14. Find relationships based on a user-defined set of available and/or text-searchable attributes or values (e.g. experiment type) – i.e. equivalent of complex/compound Booleans, with nesting and NOT. [Mane and Borner; Kang et al. (a)]

15. Understand and code by what defines a relationship (interaction/edge). [Uetz et al.]

16. Understand and code by what defines an entity (node). [Elkon et al.;Uetz et al.]

17. Have pan capabilities and geometric zoom capabilities that leave context visible. [Uetz et al.; Schrinivasan and vanDijk; Mane and Borner; Nevrasovski et al.]

18. Sort efficiently to find the concepts, measures, or attributes in the data that are already known to the user [Saraiya et al.; Kang et al. (a)]

19. Have specified layouts and/or arrangements maintained across multiple moves without reverting to some default [Mane and Borner; Faisal et al.]

20. Make focus-context transitions through perceptually guided displays – using visual techniques in the presentation space - that shift attention while maintaining prior context to ease the transition without heavy cognitive burden [Pietriga and Appert; Mane and Borner; Kang et al. (a); Yi et al.]

21. Have the ability to see subsets in the same window or a new window. [Seo and Shneiderman]

22. Standard filtering package: [Schrinivasan and van Dijk, 2008 Thomas and Cook] Users can

filter by single items (nodes, edges, attributes)

filter by 2+ attributes or values (union, intersection, subtraction)

filter by attributes assigned to a group/cluster or subgroups

specify outcomes of filtering as follows: exclude entities or relationships and update color and other coding according to items that remain; exclude entities or relationships and don't do updates, hide filtered out entities or relationships, allow hidden entities or relationships to become visible (but grayed out), allow excluded to be recalled.

see that performing filtering actions updates counts and be able to view the counts, as needed

23. Standard selection package: [Saraiya et al. (a); Wong et al.; Mirel, 2001; Seo and Shneiderman]. Users can

select single items (nodes, edges, attributes)

select 2+ items

select adjacent entities or relationships, groups/clusters or subgroups

select available fields to display

select non-adjacent entities or relationships and/or groups

select groups of entities based on 2+ self-specified attributes (e.g. specified by 2+ GO terms) and can subtract one or more of the attributes without having to redo the whole selection

select relationships based on one attribute.

find relationships based on a compound/complex user-defined set of attributes or values (e.g., equivalent of complex/compound Booleans, with nesting and NOT)

select the overlapping entities shared by 2+ clusters of interest.

select the overlapping relationships shared by 2+ networks

see that performing actions updates counts, and can view the counts as needed

24. See, choose from, and selectively hide entities or relationships either individually or based on a criterion and bring them back as needed.[Kang et al. (a); Thomas and Cook]

25. See or hide the attributes of the entities describing members of groups as needed. [Seo and Shneiderman; Dadzie and Bruger; Kang et al (a); van Eck et al]

26. Easily call up and manipulate multiple linked windows to see views side by side as needed [Seo and Shneiderman; Piringer et al.; Baldonado et al. ; Saraiya et al.; Wong et al.; Schrinivasan and van Dijk; Kang et al. (a)]

27. Save sessions and/or views – including bookmarking - as static views and as recallable states. [Gotz and Zhou; Robinson]

28. See details on group members [Kang et al. (b); da Silva Maciel et al.]

29. Sub-group within a group by a secondary or tertiary dimension [Holloway et al.]

References

Akers D, Simpson M, Jeffries R, Winograd T: Undo and erase events as indicators of usability problems. *SIGCHI Conference on Human Factors in Computing:* ACM Press; 2009:659-668

Baldonado M, Woodruff A, Kuchinsky A: Guidelines for using multiple views in information visualization. *Proceedings of the Working Conference on Advanced Visual Interfaces (AVI '00)* New York: ACM Press; 2000:110-119.

Barsky A, Gardy J, Hancock R and Munzner T: Cerebral: A cytoscape plugin for layout of and interaction with biological networks using subcellular localization annotation. *Bioinformatics* 2007, 23:1040-1042.

Brewer, C: Color use guidelines for data representation. Proceedings of the Section on Statistical Graphics, 1999:55–60,

da Silva Maciel, M, Megiuins, BS, de Moreas Lourenco, RA, Miguins, AS, Godinho, P: The impact of multiple coordinated views on the visual data exploration and analysis. *Proceedings of the 12th International Conference on Information Visualisation*. IEEE, 2008:113-119

Dadzie A-S, Burger A: Providing visualization support for the analysis of anatomy ontology data. *BMC Bioinformatics* 2005, 6:74.

Elkon R, Vesterman R, Amit N, Ulitsky I, Zohar I, Weisz M, Mass G, Orlev N et al. SPIKE – a database, visualization, and analysis tool of cellular signaling pathways. BMC Bioinformatics 9, 2008: 110-124

Elmqvist N, Hentry N, Rice Y, Fekete D:. Melange: space folding for multi-focus interaction. *SIGCHI Conference on Human Factors in Computing:* ACM Press; 2008:1333-1342

Faisal S, Craft B, Carirns P, Blandford A: Internalization, qualitative methods, and evaluation. *Proceedings of the 2008 conference on BEyond time and errors: novel evaLuation methods for Information Visualization (BeLIV).* ACM,2008:

Gotz D, Zhou MX: Characterizing users' visual analytic activity for insight provenance. *IEEE symposium on the visual analytics science and technology (VAST) 2008.* IEEE Press; 2008, 123-130

Holloway, D, Kon, M, Delisi, C: Classifying transcription factor targets and discovering relevant biological features. *Biology Direct*, 2008, 3:22.

Kang Y-A, Gorg C, Stasko J (a): Evaluating visual analytics systems for investigative analysis: deriving design principles from a case study. *IEEE Symposium on Visual Analytics Science and Technology.* IEEE, 2009:139-146

Kang,Y-A,Gorg C, Stasko J (b). How can visual analytics assist investigative analysis? Design implications from an evaluation. IEEE Transactions on Visualization and Computer Graphics (in press)

Mane K, Borner K: SRS browser: a visual interface to the sequence retrieval system. SPIE Conference on Visualization and Data Analysis 6060, 2006:1-11

Mirel, B: Testing the usability of interactive visualizations for complex problem-solving: findings related to improving interfaces and help. Journal of Technical Writing and Communication 31 (2001), 7-26

Nekrasovski, D, Bodnar, A, McGrenere, J, Guimbretiere, Munzer, T: An evaluation of pan & zoom and rubber sheet navigation with and without an overview. Proceedings of the SIGCHI Conference on Human Factors in Computing (CHI 06), 2006, 11-20

Perer A, Shneiderman B: Integrating statistics and visualization: case studies of gaining clarity during exploratory data analysis. *SIGCHI Conference on Human Factors in Computing:* ACM Press; 2008:265-274

Pietriga E, Appert C: Signma lenses: focus-context transitions combining space, time, and translucence. Proceedings of the 26th annual SIGCHI conference on human factors in computing systems. New York: ACM, 2008: 1343-1352

Piringer H, Berger W, Hauser H: Quantifying and comparing features in high-dimensional datasets. *Proceedings of the 12th International Conference on Information Visualisation*. IEEE, 2008:240-245

Robinson A: Needs assessment for the design of information synthesis visual analytics tools. *Proceedings of the 13th International Conference Information Visualisation*. IEEE, 2009, 353-360

Saraiya P,, North C, Duca K: An insight-based methodology for evaluating bioinformatics visualizations. *IEEE Transactions on Visualization and Computer Graphic*s, 2005; 11:443-456

Schrinivasan Y, Van Wijk J: Supporting the analytical process with visualizations. *Proceedings of the 26th Annual SIGCHI Conference on Human Factors in Computing 2008*: ACM Press; 2008:1237-1246.

Seo J, Shneiderman B: Knowledge discovery in high dimensional data: case studies and a user survey for an information visualization tool. *IEEE Transactions on Visualization and Computer Graphics* 2006, 12:311-322

Thomas, JJ, Cook, KA: *Illuminating the path*. Richland, WA: National Visualization and Analytics Center, 2005

Tominski C, Fuchs G, Schumann H: Task-driven color coding. *Proceedings of the 12th International Conference on Information Visualisation*. IEEE, 2008:373-380

Uetz P, Ideker T, Schwikowski B: Visualization and integration of protein-protein interactions**.** In *The Study of Protein-Protein Interactions- An Advanced Manual*. Edited by Golemis E: Woodbury,NY: Cold Spring Harbor Laboratory Press, 2005.

Van Eck NJ, Frasincar F, Chang D: Cluster-based visualization of concept associations. *Proceedings of the 12th International Conference on Information Visualisation*. IEEE, 2008:409-414

Wong PC, Foote H, Chin Jr. G, Mackey P, Perrine K: Graph signatures for visual analytics**.** *IEEE Transactions on Visualization and Computer Graphics* 2006, 12:1399-1413.

Yi, JS, Kang, Y-A, Stasko, J, Jacko, J: Understanding and characterizing insights: how do people gain insights using information visualization? *Proceedings of the 2008 conference on BEyond time and errors: novel evaLuation methods for Information Visualization (BeLIV).* ACM, 2008: 1-6
